# Unveiling the Enigma of a Colonic Neuroendocrine Tumor Causing Ileocolic Intussusception: A Case Report

**DOI:** 10.7759/cureus.54823

**Published:** 2024-02-24

**Authors:** Natalie Mesa, Lizis O Rodriguez, Mitchel Lacey, Rupa Seetharamaiah

**Affiliations:** 1 General Surgery, Florida International University, Herbert Wertheim College of Medicine, Miami, USA; 2 Surgery, Florida International University, Herbert Wertheim College of Medicine, Miami, USA; 3 Department of Surgery, Baptist Hospital of Miami, Miami, USA

**Keywords:** bowel obstruction, abdominal pain, hemicolectomy, general surgery, adult intussusception, colonic neuroendocrine tumor

## Abstract

Intussusception in adults is rare and is often associated with a pathologic lead point. While colonic adenocarcinoma is a common cause, well-differentiated colonic neuroendocrine tumors are exceedingly rare. We present a unique case of an ileocolic intussusception due to a distal ascending colonic neuroendocrine tumor, emphasizing the diagnostic challenges and importance of prompt intervention.

A 60-year-old male with a previous screening colonoscopy in June of 2022 presented to the Emergency Department with two days of cramping, right upper abdominal pain with associated nausea and two episodes of emesis. A Computed Tomography (CT) scan of the abdomen and pelvis revealed an ileocolic intussusception noted at the level of the hepatic flexure with a lead point. Emergent surgical intervention identified a mass in the distal ascending colon, and a right hemicolectomy with successful side-to-side functional end-to-end anastomosis was performed. Final pathology confirmed a well-differentiated stage III colonic neuroendocrine tumor. After a successful postoperative recovery, a full body Positron Emission Tomography (PET) scan was completed and resulted in no evidence of avid metastatic disease. The patient was placed in cancer remission.

Intussusceptions in the adult population are uncommon, and the etiology typically involves a pathologic lead point causing intestinal invagination. In this case, prompt diagnosis and management resulted in successful health outcomes with reduced mortality and morbidity, as untreated intussusception can have devastating results. Given this patient’s colonoscopy was approximately one year ago, the probability of a colonic neoplasm acting as the lead point was low. However, identification of the intussusception resulted in a timely and lifesaving emergent right hemicolectomy, as this stage III tumor has a five-year median survival rate of only 50% if left untreated. This case report highlights a rare case of adult ileocolic intussusception involving a lead point at the distal ascending colon identified as an uncommon, well-differentiated stage III neuroendocrine tumor. It showcases the importance of considering intussusception as a diagnosis when evaluating adults with abdominal pain for prompt and adequate intervention, especially when malignant lead points and bowel necrosis are suspected.

## Introduction

Intussusception is known as the invagination, or telescoping, of a portion of the intestine into itself. In children, it is the leading cause of intestinal obstruction and is considered an abdominal emergency [1]. This mechanism of mechanical intestinal obstruction is a rare occurrence in adults, with it accounting for only 5% of all intussusceptions, 1% of all bowel obstructions, and 0.003% to 0.02% of hospital admissions [2]. Etiology varies amongst children and adults, with about 90% of adults having a pathologic lead point, while occurrences in children are mostly idiopathic; only 25% of cases in children involve an aberrant focal point [1,3]. Pathologic lead points are more commonly found to originate from the small bowel, accounting for 66.7% of adult intussusceptions, while colonic lead points account for only 12.5% of the cases [4,5]. When compared to the small bowel, the higher prevalence of colon cancer results in an increased frequency of malignant lead points, most commonly primary colonic adenocarcinoma. On the other hand, lipomas have been found to be the most common benign colonic focal point [6].

Neuroendocrine tumors (NETs) consist of neuroendocrine cells, which are mixed cells that receive signals from neurons and emit hormones and neurotransmitters in response [7]. Although approximately two-thirds of NETs emerge from the gastrointestinal system, less than 0.01% arise in the colon [8]. Symptoms at presentation are clinically similar to those of colonic adenocarcinoma, but patients diagnosed with colonic neuroendocrine tumor (C-NET) typically have a more advanced stage at diagnosis [9]. 

In this report, we present a rare case involving a 60-year-old male diagnosed with an ileocolic intussusception extending to the hepatic flexure, with a lead point identified as a well-differentiated neuroendocrine tumor localized to the distal ascending colon. Subsequently, the patient underwent emergent resection of the right colon with successful anastomosis between the distal ileum and transverse colon. Our case exhibits a rare presentation of intussusception in an adult and the discovery of a malignant C-NET. This case report was written per the Surgical Case Report (SCARE) Guidelines [10].

## Case presentation

In August of 2023, a 60-year-old male with a past medical history of hypertension, obstructive sleep apnea, aortic regurgitation, hyperlipidemia, and coronary artery calcification presented to the emergency department (ED) with complaints of upper abdominal pain for two days. The patient presented with six out of 10 cramping right upper quadrant pain (RUQ) with associated nausea, two episodes of emesis, and over five episodes of diarrhea. The patient mentioned relief of abdominal pain after vomiting. He denied any fevers, chills, shortness of breath, hematemesis, melena, or hematochezia at that time. The patient denied any recent travel, antibiotic use, or sick contacts. Past surgical history includes bilateral knee meniscectomy with no reported abdominal surgeries. His last colonoscopy was completed in June 2022, and benign polyps were noted at the time, as per the patient. A previous endoscopy was completed 10 years ago with no significant findings.

On presentation in the ED, the patient was afebrile and normotensive. Abdominal examination revealed that the abdomen was soft, tympanic to percussion, and tender to palpation in the RUQ with no rigidity or guarding. Laboratory tests at that time revealed a normal WBC of 7.6 K/uL, hemoglobin of 17.2 g/dL, mildly elevated hematocrit of 51.1%, and a normal platelet count of 208 K/uL. He was noted to have normal electrolytes, liver function tests, and lactic acid of 0.9 mmol/L. 

**Figure 1:**
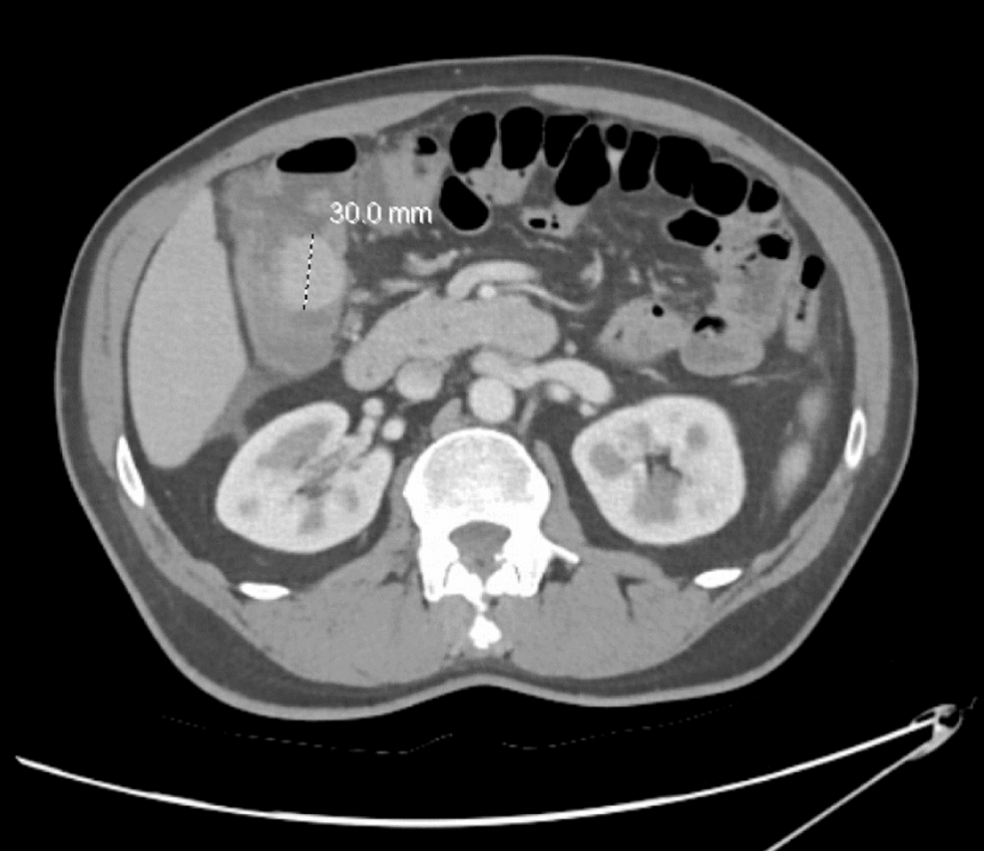
CT of the abdomen and pelvis with intravenous contrast, axial view.

**Figure 2:**
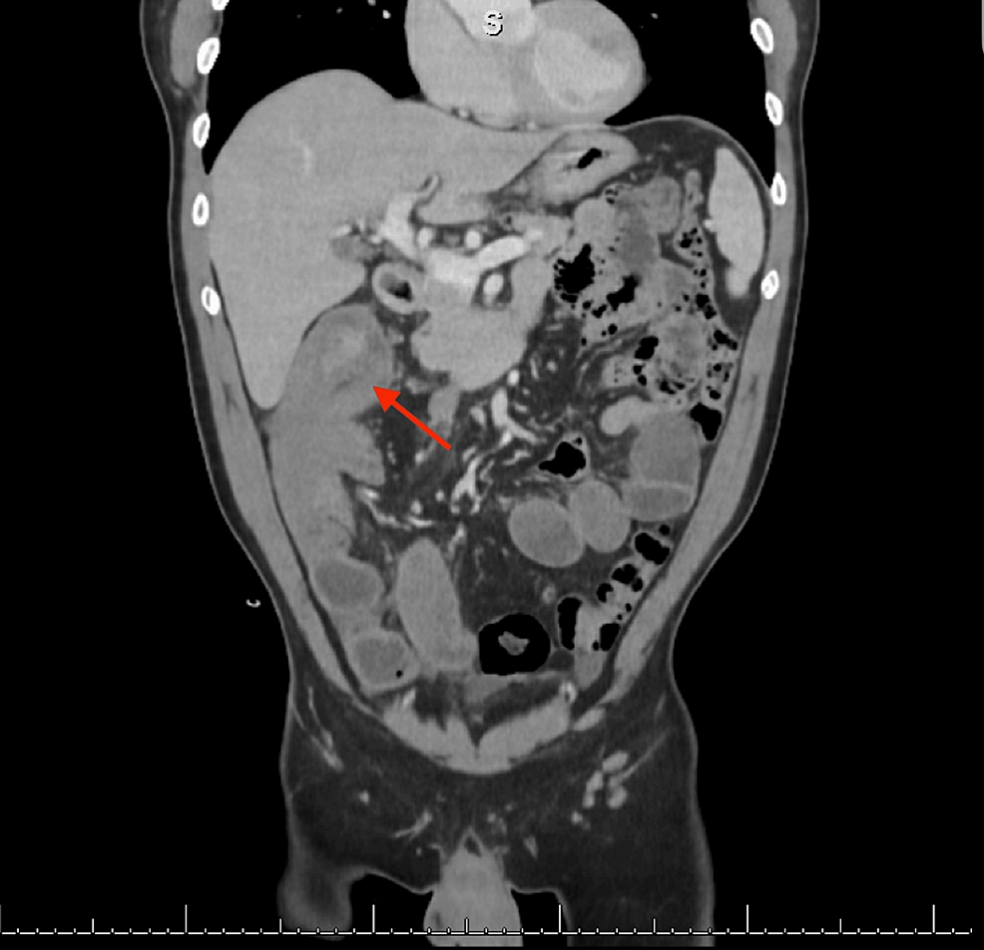
CT of the abdomen and pelvis with intravenous contrast, sagittal view.

A computed tomography (CT) scan of the abdomen and pelvis with intravenous contrast revealed an ileocolic intussusception noted at the level of the hepatic flexure with a lead point measuring 3.0 cm, likely related to a neoplasm (Figures 1, 2). Dilation of fluid-filled mid-to-distal small bowel loops with adjacent perienteric fat stranding in the right mid and lower quadrant of the mesentery was noted. These findings indicated a high-grade small bowel obstruction (SBO) with possible bowel compromise. Furthermore, there was a 1.0 cm mildly enhancing lesion in segment seven of the liver and an enlarged lymph node measuring 1.7 cm in the mesentery.

**Figure 3:**
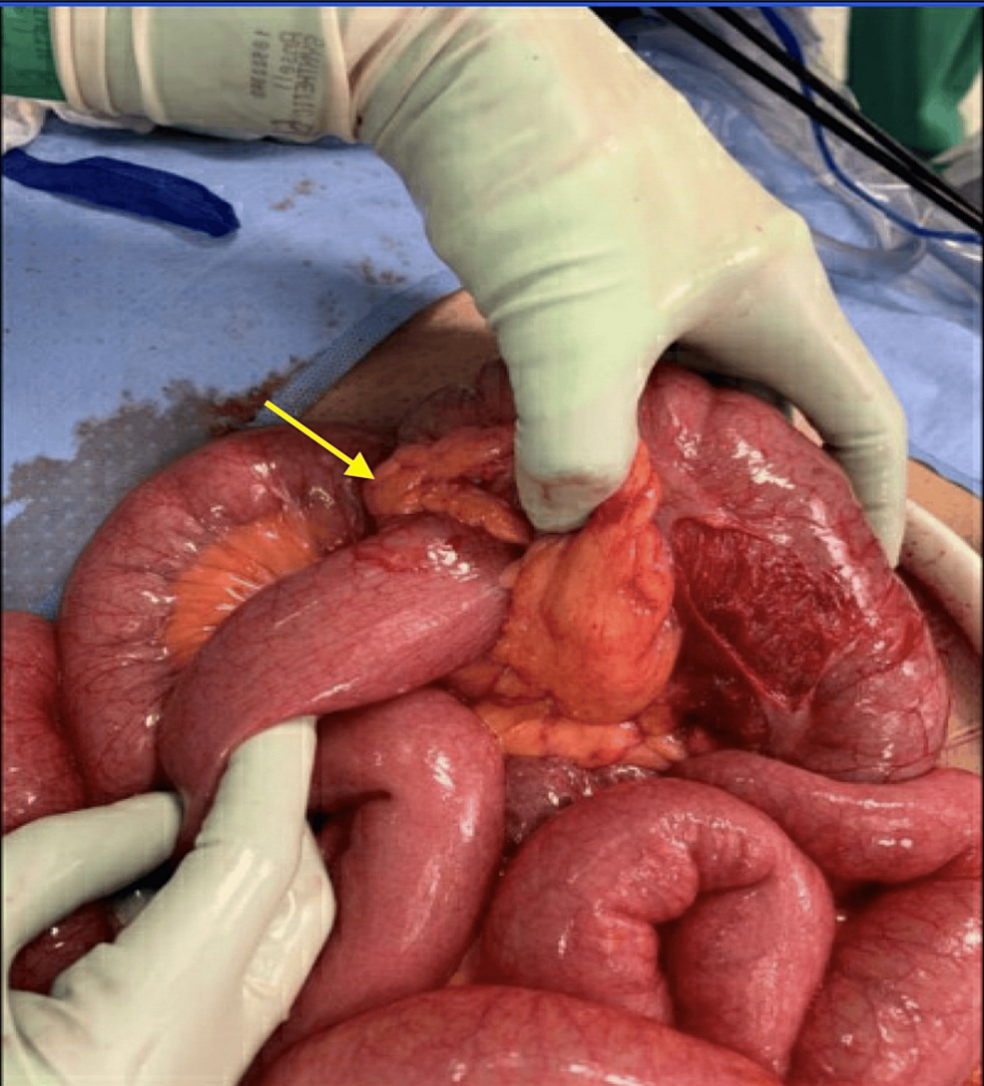
Image of the resected bowel specimen The arrow shows the Ileocolic intussusception causing small bowel obstruction with visualization of the distal terminal ileum.

Given the high suspicion of small bowel compromise, general surgery was consulted, and an emergent diagnostic laparoscopy was performed within hours of admission. Intraoperatively, an ileocolic intussusception was noted extending to the hepatic flexure. After complete mobilization of the hepatic flexure, two large, hard nodules were noted to be attached posterior to the duodenum. Thus, an exploratory laparotomy was performed for safe resection of the mass. A right hemicolectomy with distal small bowel resection was completed followed by a successful side-to-side functional end-to-end anastomosis between the distal ileum and transverse colon. Gross examination of the resected bowel specimen revealed telescoping of the distal terminal ileum into the cecum and ascending colon up to the hepatic flexure (Figure 3).

**Figure 4:**
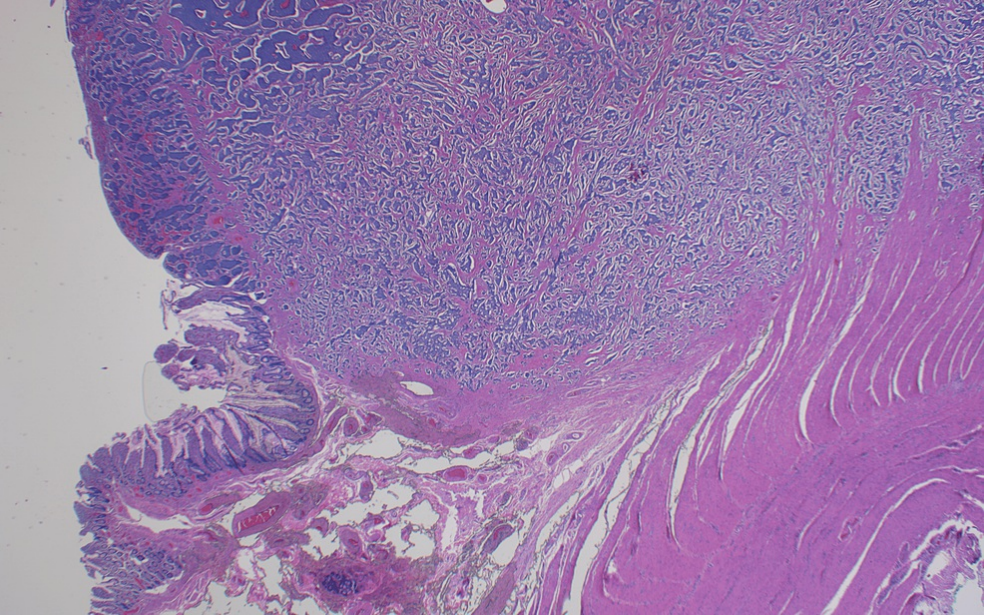
Histopathology image of the neuroendocrine tumor (upper half) involving the colonic tissue

**Figure 5:**
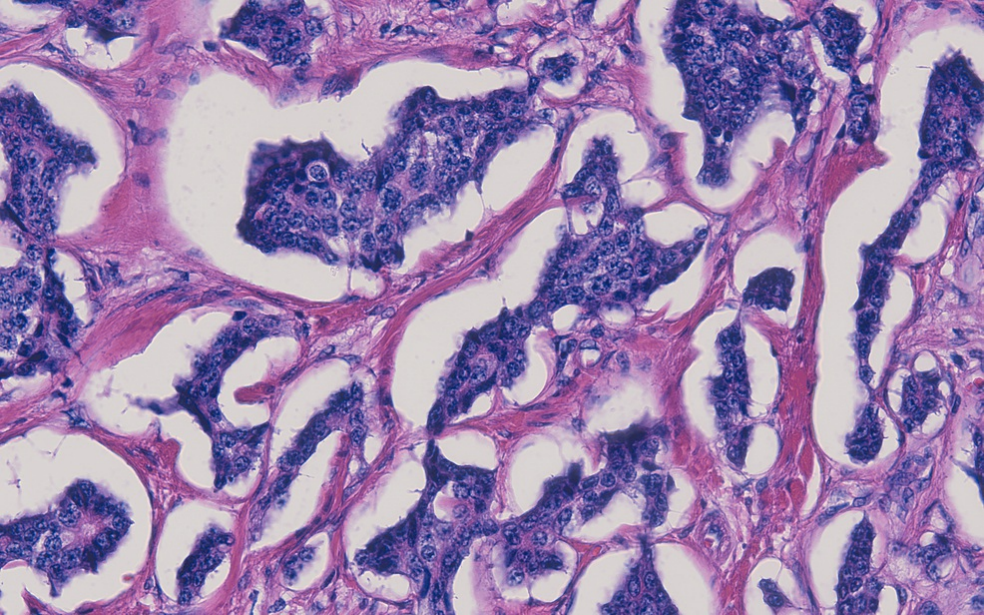
Histopathological study of the neuroendocrine tumor The image shows the neuroendocrine tumor with a characteristically described salt and pepper chromatin pattern.

**Figure 6:**
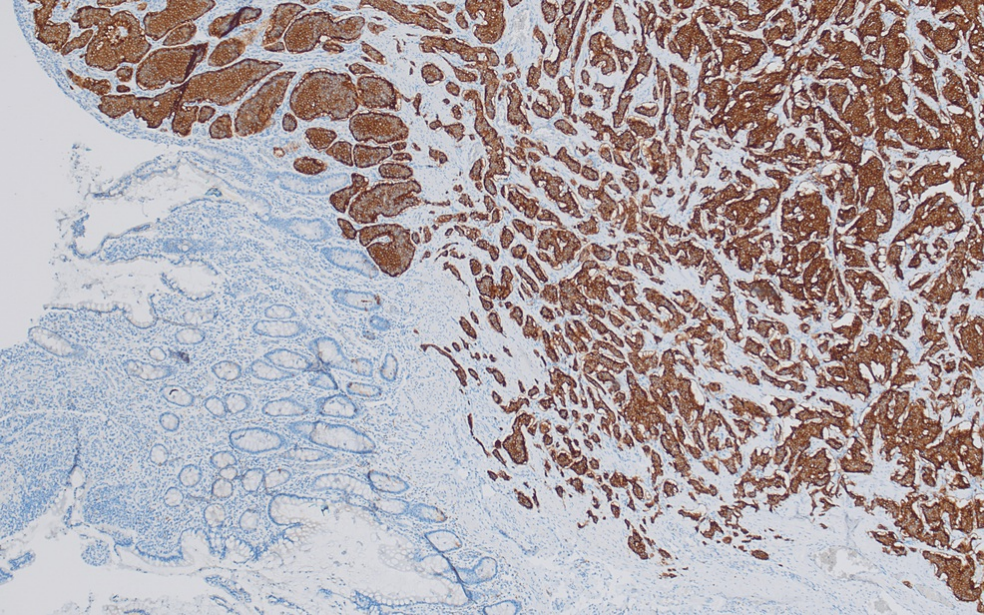
Positive synaptophysin immunostain of colonic tissue

The liver was then examined and no gross masses or lesions were observed. Bowel specimens with adjacent lymph nodes were sent to pathology for further analysis. The surgical pathology report showed a 2.8 x 2.5 cm mass localized to the distal ascending colon, revealing a well-differentiated neuroendocrine tumor staged T3N1M0, grade 1, and positive for synaptophysin, chromogranin, INSM1, and keratin AE1/AE3 (Figures 4-6). Proximal and distal specimen margins were tumor-free, and two out of 22 resected lymph nodes were found to have metastatic carcinoma. Abdominal fluid cytology collected intraoperatively was negative for malignant cells. Chest CT scan completed post-operatively was negative for metastatic disease.

The patient had a successful recovery and was sent home in stable condition on post-operative day six. He completed recommended follow-up visits with the surgeon and was noted to be progressing well. The patient was referred to a medical oncologist for further management. A full-body positron emission tomography (PET) scan was ordered to rule out metastatic disease. Results revealed no evidence of Gallium-68 avid metastatic disease, and the patient was placed in cancer remission with close follow-up care. The patient gave informed consent for the publication of this case report.

## Discussion

Intussusceptions in the adult population are uncommon and etiology typically involves a pathologic lead point causing intestinal invagination. Literature has shown that many of these focal points arise in the small bowel, with very few cases localized in the colon, the most common colonic pathology being colonic adenocarcinoma [4-6]. Well-differentiated C-NETs make up 5-7% of all NETs in the gastrointestinal tract. When compared to rectal NETs, they are known to have a worse prognosis and have the lowest survival rate of all NETs. For stage III NETs, the median survival rate is 50% [9]. This case report highlights a rare case of adult ileocolic intussusception involving a lead point at the distal ascending colon identified as an uncommon, well-differentiated neuroendocrine tumor stage III at the time of diagnosis. The patient’s recent colonoscopy suggested no indication of a possible lead point, contributing to the rarity of this case. 

The standard of treatment for adult intussusception is en bloc surgical resection, especially when bowel ischemia is suspected or if there is increased suggestion of malignant etiology. To optimize surgical and clinical outcomes in these patients, oncological principles should be followed, including lymphadenectomy, obtaining at least 12 lymph nodes for adequate prognosis and post-operative treatment plan [6]. These principles were followed with our patient and were vital to the diagnostic process and development of a patient-centered treatment strategy. With this method, it is important to consider the location of the intussusception and suspicion of bowel necrosis and malignancy; however, the addition of a less invasive treatment option expands the alternatives to surgery. 

Abdominal pain can be considered one of the most common presenting symptoms in the ED, serving as a manifestation of numerous gastrointestinal pathologies. This case showcases the importance of considering intussusception as a diagnosis when evaluating adults with abdominal pain for prompt and adequate intervention. Proper treatment management in cases like these will prevent morbidity and mortality, as untreated intussusception can have devastating outcomes, especially when malignant lead points and bowel necrosis are suspected. Given the patient had his most recent colonoscopy one year ago, the probability of a colonic neoplasm acting as the lead point of intussusception was lower on the list of differentials. However, the identification of his symptomatic intussusception incidentally resulted in the discovery of a colonic neuroendocrine tumor, which was a rare occurrence given the patient’s recent appropriate preventative screening for colon cancer. These findings resulted in a timely and lifesaving emergent surgical intervention, as his stage III tumor has a five-year median survival rate of only 50% if left untreated [9].

## Conclusions

Our case unravels the complexity of a C-NET serving as an unexpected lead point for ileocolic intussusception in a 60-year-old male. Adult intussusception is a rare entity and our patient’s diagnosis adds to the limited literature on such occurrences and emphasizes the importance of increasing one’s index of suspicion for rare differentials when evaluating abdominal pain of unknown etiology.

This case contributes to the evolving understanding of adult intussusception, emphasizing the need for a comprehensive diagnostic approach, even in the absence of typical risk factors. Notably, the incidental discovery of this malignancy, despite the patient’s recent colonoscopy, highlights the diagnostic challenges associated with these rare presentations. As our understanding of such cases grows, future research may unveil additional nuances to the diagnosis and management of adult intussusception with uncommon pathologic lead points.
